# Broad-spectrum antibiotics disrupt homeostatic efferocytosis

**DOI:** 10.1038/s42255-024-01107-7

**Published:** 2024-08-09

**Authors:** Pedro H. V. Saavedra, Alissa J. Trzeciak, Allie Lipshutz, Andrew W. Daman, Anya J. O’Neal, Zong-Lin Liu, Zhaoquan Wang, Jesús E. Romero-Pichardo, Waleska Saitz Rojas, Giulia Zago, Marcel R. M. van den Brink, Steven Z. Josefowicz, Christopher D. Lucas, Christopher J. Anderson, Alexander Y. Rudensky, Justin S. A. Perry

**Affiliations:** 1 Immunology Program, Sloan Kettering Institute, Memorial Sloan Kettering Cancer Center, New York, NY, USA; 2 Immunology and Microbial Pathogenesis Program, Weill Cornell Medical College, Cornell University, New York, NY, USA; 3 Department of Pathology and Laboratory Medicine, Weill Cornell Medical College, Cornell University, New York, NY, USA; 4 Louis V. Gerstner Jr. Graduate School of Biomedical Sciences, Memorial Sloan Kettering Cancer Center, New York, NY, USA; 5 Adult Bone Marrow Transplantation Service, Department of Medicine, Memorial Sloan Kettering Cancer Center, New York, NY, USA; 6 Department of Medicine, Weill Cornell Medical College, Cornell University, New York, NY, USA; 7 University of Edinburgh Centre for Inflammation Research, Queen’s Medical Research Institute, Edinburgh BioQuarter, UK; 8 Institute for Regeneration and Repair, Edinburgh BioQuarter, UK; 9 Howard Hughes Medical Institute and Ludwig Center, Memorial Sloan Kettering Cancer Center, New York, NY, USA

## Abstract

The clearance of apoptotic cells, termed efferocytosis, is essential for tissue homeostasis and prevention of autoimmunity^1^. Although past studies have elucidated local molecular signals that regulate homeostatic efferocytosis in a tissue^2,3^, whether signals arising distally also regulate homeostatic efferocytosis remains elusive. Here, we show that large peritoneal macrophages (LPMs) display impairs efferocytosis in broad-spectrum antibiotics (ABX)-treated, vancomycin-treated, and germ-free mice in vivo, all of which have a depleted gut microbiota. Mechanistically, the microbiota-derived short-chain fatty acid butyrate directly boosts efferocytosis efficiency and capacity in mouse and human macrophages, and rescues ABX-induced LPM efferocytosis defects in vivo. Bulk mRNA sequencing of butyrate-treated macrophages in vitro and single cell mRNA sequencing of LPMs isolated from ABX-treated and butyrate-rescued mice reveals regulation of efferocytosis-supportive transcriptional programs. Specifically, we find that the efferocytosis receptor T-cell immunoglobulin and mucin domain containing 4 (TIM-4, Timd4) is downregulated in LPMs of ABX-treated mice but rescued by oral butyrate. We show that TIM-4 is required for the butyrate-induced enhancement of LPM efferocytosis capacity and that LPM efferocytosis is impaired beyond withdrawal of ABX. ABX-treated mice exhibit significantly worse disease in a mouse model of lupus. Our results demonstrate that homeostatic efferocytosis relies on distal metabolic signals and suggest that defective homeostatic efferocytosis may explain the link between ABX use and inflammatory disease^4–7^.

## Main

Over 1% of our cellular biomass, approximately 3x10^11^ cells, is turned over every day^1^. The majority of this cell turnover is performed by phagocytes embedded in tissues, such as tissue-resident macrophages, through a process termed homeostatic efferocytosis^2,3,8^. Indeed, failure to efficiently clear ACs via homeostatic efferocytosis is associated with the onset and progression of numerous autoimmune and inflammatory diseases^2,3,9,10^. Understanding how metabolic signals generated outside of a given tissue (‘distal signals’) regulate a phagocyte’s efferocytosis potential remains underexplored^11^. Given the importance of the intestinal microbiome to host homeostasis, its known role in regulating the immune system distally, and the direct link between broad-spectrum antibiotics-induced dysbiosis and onset of diseases associated with impaired homeostatic efferocytosis, we reasoned that the molecular signals produced by the microbiome regulate homeostatic efferocytosis. Here, we find that large peritoneal macrophages (LPMs) display impaired efferocytosis in mice treated with broad-spectrum antibiotics (ABX). We find that the microbiota-derived short-chain fatty acid (SCFA) butyrate directly boosts efferocytosis in mouse and human macrophages and rescued ABX-induced LPM efferocytosis defects *in vivo*. We report that butyrate regulates efferocytosis-supportive transcriptional programs *in vitro* and *in vivo*. Specifically, we found that the efferocytosis receptor T-cell immunoglobulin and mucin domain containing 4 (TIM-4, *Timd4*) was downregulated in LPMs of ABX-treated mice but rescued by oral butyrate and accounted for the butyrate-induced enhancement of LPM efferocytosis capacity. Finally, ABX-treated mice displayed worse disease in a mouse model of lupus. Our results demonstrate that homeostatic efferocytosis relies on distal metabolic signals and suggest that defective homeostatic efferocytosis may explain link between ABX use and inflammatory disease.

**Extended Data Fig. 1:**
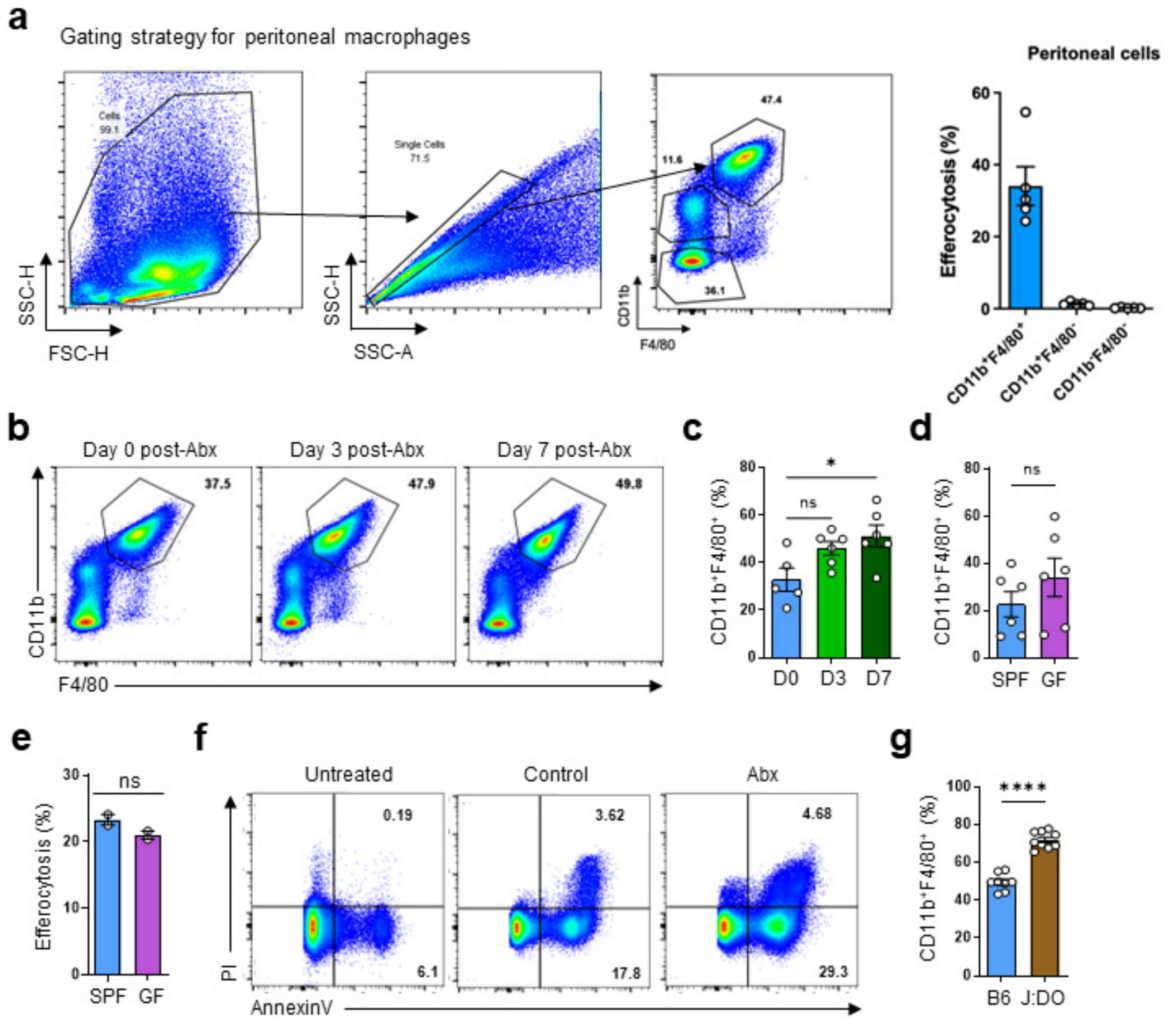


**Fig. 1:**
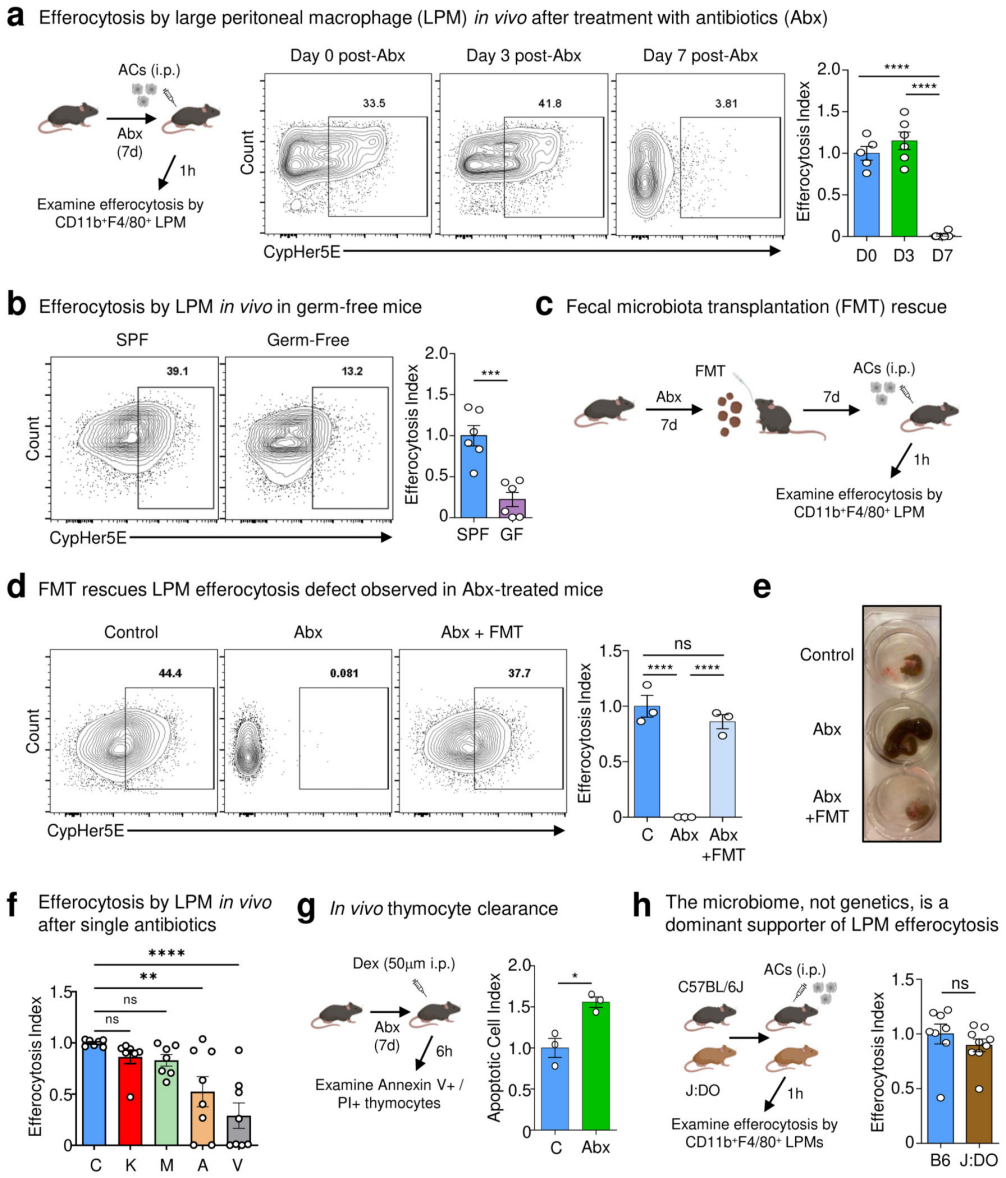
The intestinal microbiome supports peripheral efferocytosis. (a) Mice were treated with antibiotics in drinking water for 0-7d prior to intraperitoneal (i.p.) injection of CypHer5E-labeled apoptotic cells (ACs). After 1h, LPMs (CD11b^+^F4/80^+^) were analyzed for efferocytosis efficiency. Shown are efferocytosis efficiency by LPMs in untreated mice (D0, n = 5), in mice after 3d of broad-spectrum antibiotics (D3, n = 6), and in mice after 7d antibiotics (D7, n = 6). Data are from two independent experiments with between two to three mice per experiment. (b) *In vivo* efferocytosis as in (a), but in specific pathogen-free (SPF) (n = 6) and germ-free (GF) (n = 6) mice. Data are from two independent experiments with three mice per experiment. (c-e) Mice were treated with antibiotics in drinking water for 7d, with some mice receiving fecal microbiota transplantation (FMT) of homogenized stool from SPF mice (c). (d) Efferocytosis efficiency by LPMs in control mice (n = 3), in broad-spectrum antibiotic-treated mice (ABX, n = 3), and in broad-spectrum antibiotic-treated mice supplemented with FMT (ABX+FMT, n = 3). (e) Representative images of the cecum of mice from (d). Data are from two independent experiments with between one to two mice per experiment. (f) Efferocytosis efficiency by LPMs in control (C; n = 7), kanamycin (K; n = 7), metronidazole (M; n=7), ampicillin (A; n = 8), or vancomycin (V; n = 8) treated mice. Data are from two independent experiments with between three to four mice per experiment. (g) AnnexinV^+^PI^+^ staining from isolated thymocytes 6h post-dexamethasone (Dex) injection in vehicle (n = 3) or antibiotic-treated mice (n = 3). Data are from three experimental replicates. (h) Efferocytosis by LPMs in C57BL6/J (B6, n = 8) and J:DO (n = 9) mice. Data are from two independent experiments with between four to five mice per experiment. All bar graphs represent means ± s.e.m. Statistics were performed by one-way ANOVA in **a, d**, and **f**, two-tailed *t*-test in **b, g** and **h**. *p < .05; **p < .01; ***p < .001. ****p < .0001. ns - not significant. Schematics were created with BioRender.com.

To investigate whether healthy intestinal microbiota informs the ability of tissue-resident macrophages (TRMs) to perform homeostatic efferocytosis, we focused on large peritoneal macrophages (LPMs), a highly phagocytic, long-lived resident TRM population with direct access to circulating substrates^12^. We treated mice with an oral course of broad-spectrum antibiotics (ABX) prior to peritoneal injection of apoptotic cells (ACs) *in vivo* (Fig. 1a). Analysis of peritoneal exudates revealed that resident LPMs (CD11b^+^ F4/80^+^; Extended Data Fig. 1a), the phagocyte primarily responsible for homeostatic efferocytosis in the peritoneum^13^, exhibit dramatically decreased ability to internalize and digest ACs in mice treated with a 7d course of ABX (Fig. 1a), corresponding to the timepoint at which the majority of intestinal bacteria are removed^14^. As an alternative approach to ABX, we performed peritoneal homeostasis efferocytosis assays with germ-free (GF) mice. Compared to specific-pathogen-free (SPF) mice, LPMs in GF mice exhibited significantly decreased efferocytosis capacity (Fig. 1b), without significantly decreasing LPM numbers (Extended Data Fig. 1b-d). Thus, mice treated with broad-spectrum antibiotics exhibit defective homeostatic efferocytosis in a non-intestinal tissue.

To directly test the requirement of intestinal microbes on homeostatic efferocytosis in the peritoneum, we reconstituted the microbiomes of ABX-treated mice via fecal microbiome transplantation (FMT) (Fig. 1c). ABX-treated mice reconstituted via FMT exhibited restored homeostatic efferocytosis capacity (Fig. 1d) and concomitant reversal of the enlarged caecum observed in ABX-treated mice (Fig. 1e), suggesting efficient intestinal microbiome recolonization. Our finding that homeostatic efferocytosis by LPMs was restored in FMT recipients suggested that factors originating from intestinal microbes continually educate resident LPMs instead of imprinting multipotent myeloid progenitors or hematopoietic stem cells, as has been observed in different contexts^15,16^. In support of the former, we found that primary bone marrow-derived macrophages (BMDMs; referred as macrophages) isolated from GF and SPF mice exhibited equivalent efferocytosis capacity (Extended Data Fig. 1e). Additionally, we queried whether the effect of ABX treatment depended on the presence of each individual antibiotic in the cocktail or if individual antibiotics could reproduce the phenotype, we next tested a 7d course of each antibiotic. Further, treatment with vancomycin, and to a lesser extent ampicillin, resulted in a significant decrease in LPM efferocytosis capacity, whereas kanamycin and metronidazole had no effect (Fig. 1f).

Next, we sought to determine if the defect in homeostatic efferocytosis observed in the peritoneum extends to other tissues. To this end, we used the dexamethasone-induced thymocyte cell death model to test homeostatic efferocytosis in the thymus. Similar to our findings in the peritoneum, we found that ABX-treated mice exhibited significantly increased apoptotic thymocytes, suggesting decreased thymic macrophage efferocytosis capacity (Fig. 1g and Extended Data Fig. 1f).

We next queried whether microbiome-derived signals were a dominant factor informing peripheral efferocytosis capacity by LPMs. To test this, we took advantage of the Jackson Laboratory-maintained Diversity Outbred (J:DO) strains, which share similar microbiomes with JAX-obtained C57BL/6J mice but exhibit heterogeneous genomes across individuals^17^. We housed J:DO and age- and sex-matched C57BL/6J mice in our animal facility for ~4 weeks prior to performing peritoneal homeostatic efferocytosis assays to further normalize microbial composition. We observed similar homeostatic efferocytosis capacity between J:DO and C57BL/6J mice (Fig. 1h), despite the observation that J:DO mice have an increased fraction of LPMs (Extended Data Fig. 1g). Because our *in vivo* efferocytosis assay is performed with saturating numbers of ACs, an elevation in macrophage frequency is unlikely to confound the interpretation of the current experiment. However, in tissues or contexts where AC numbers are limiting or sparsely distributed, an abundance of mature TRMs may provide an added layer of protection against a breakdown in homeostasis. Nevertheless, our findings suggest that intestinal microbe-derived factors are a dominant signal informing homeostatic efferocytosis by TRMs in non-intestinal tissues.

**Extended Data Fig. 2:**
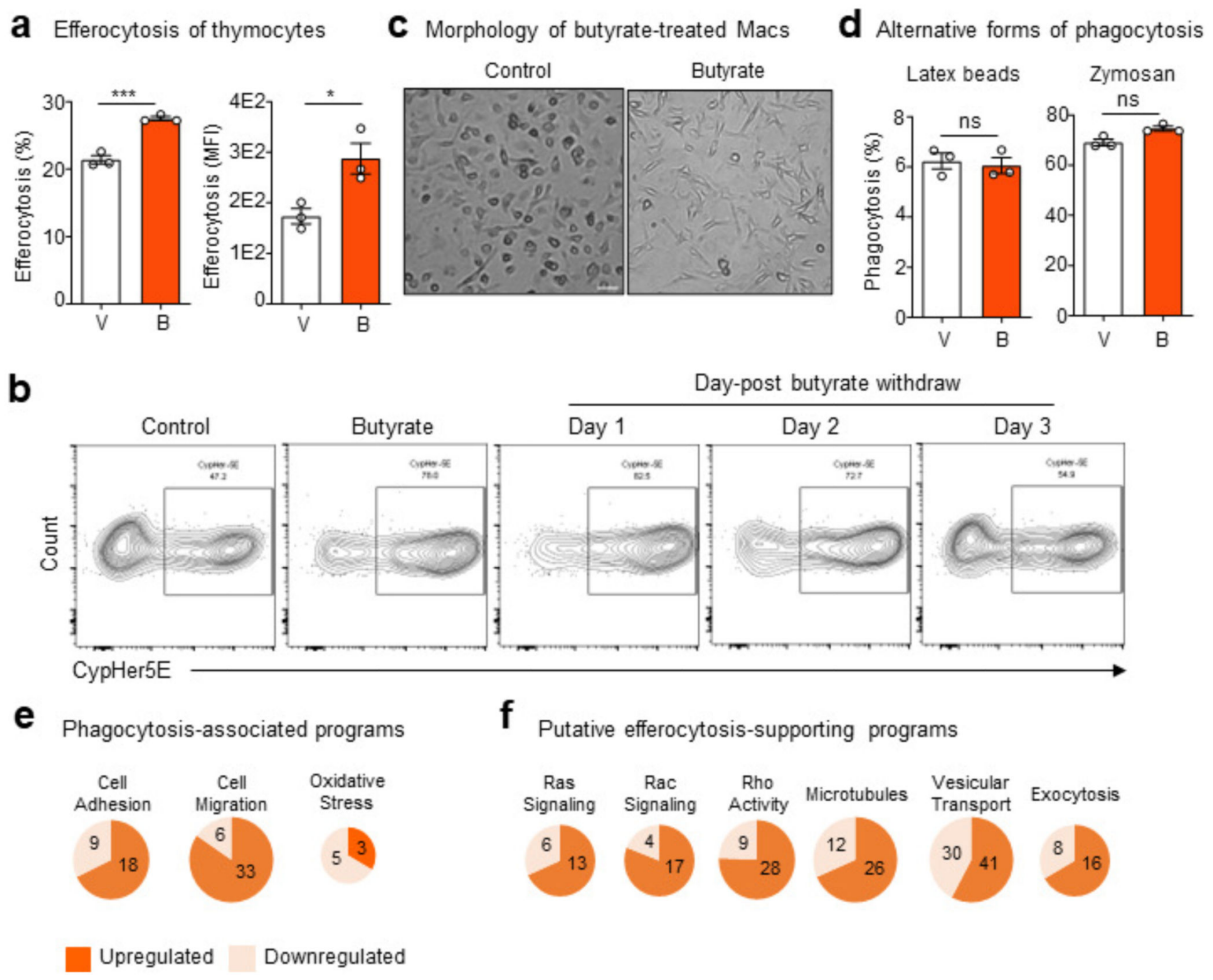


**Fig. 2:**
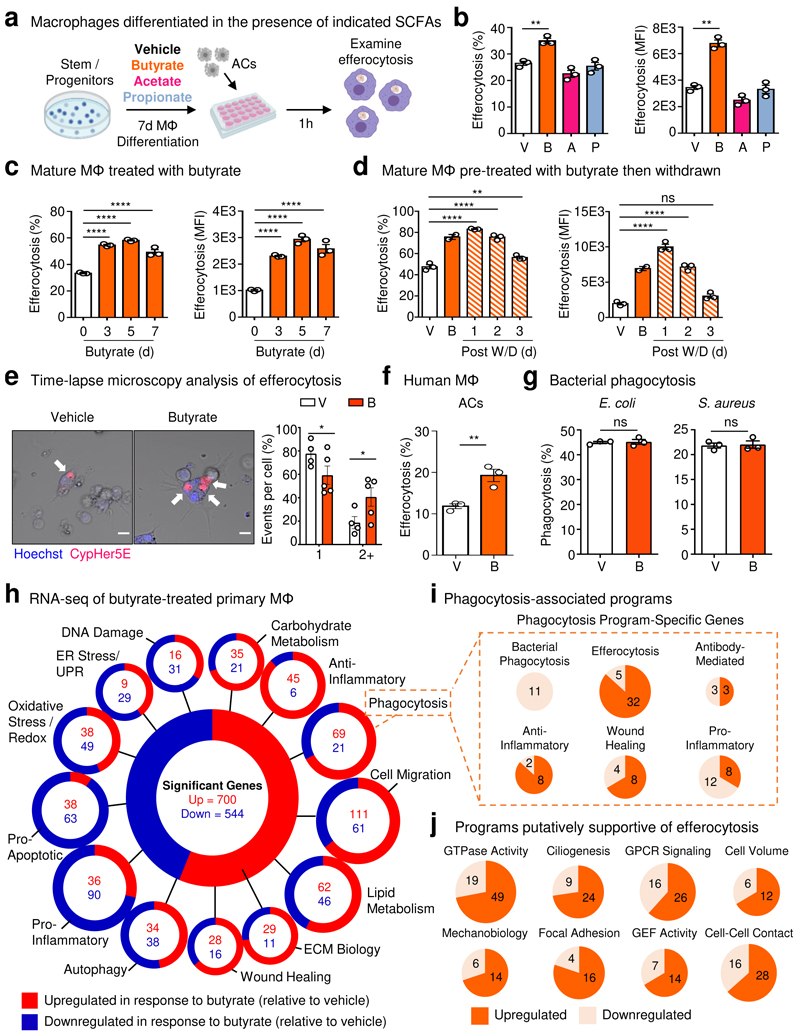
Butyrate boosts efferocytosis via induction of efferocytotic transcriptional programs. (a, b) Bone marrow was differentiated into macrophages in the presence of butyrate, acetate, or propionate (all 1 mM) for 7d (a). Mature macrophages were subsequently incubated with apoptotic cells co-labeled with CypHer5E and CellTrace Yellow (CTY) at a 1:1 ratio for 1h. (b) Efferocytosis efficiency (left, CypHer5E+ macrophages) and capacity (right, CTY median fluorescence intensity, MFI in macrophages). Data are from three independent experiments. (c) Efferocytosis efficiency of mature primary macrophages (left, CypHer5E+ macrophages) and capacity (right, CTY MFI in macrophages). Data are from three independent experiments. (d) Efferocytosis efficiency (left, CypHer5E+ macrophages) and capacity (right, CTY MFI in macrophages) followed by withdrawal of butyrate. Data are from three independent experiments. (e) Time-lapse confocal microscopy analysis of primary macrophages conditioned as in (c) then cultured with CypHer5E-labeled apoptotic cells. Macrophages containing at least one CypHer5E puncta (white arrows) were analyzed for the number of apoptotic cell uptake (CypHer5E+) events occurring. Data is shown as the fraction of the total number of CypHer5E+ events. Scale bar, 10 μm. Data are from three independent experiments. (f) Efferocytosis efficiency by human monocyte-derived primary macrophages (HMDMs) treated with vehicle or butyrate (1 mM) for 3d. Data are from three independent experiments. (g) Phagocytosis efficiency of *E. coli* (left) or *S. aureus* (right) by macrophages treated with vehicle or butyrate (1 mM) for 3d. Data are from three independent experiments. (h) RNA sequencing of mature primary macrophages conditioned with vehicle or butyrate (1 mM) for 3d. Data are from three independent experiments. ECM, extracellular matrix. (i,j) Transcripts that were categorized as ‘phagocytosis’ in (h) were further analyzed for additional functional signatures, including specific forms of phagocytosis (i) and putative efferocytosis-associated programs (j). All bar graphs represent means ± s.e.m. Statistics were performed by one-way ANOVA in **b, c**, and **d**, two-tailed *t*-test in **e, f** and **g**. *p < .05; **p < .01; ***p < .001. ****p < .0001. ns - not significant. Schematics were created with BioRender.com.

Given our finding that ABX, especially vancomycin, impairs homeostatic efferocytosis by LPMs and that microbiota-derived metabolites such as short-chain fatty acids (SCFAs) regulate immune cell function locally^18^ and possibly peripherally^19^, we sought to determine if SCFAs affect efferocytosis. To this end, we first cultured bone marrow progenitor cells with each of the SCFAs, butyrate, acetate, or propionate, during macrophage differentiation (Fig. 2a). Treatment with butyrate, but not acetate or propionate, markedly increased efferocytosis efficiency and capacity (Fig. 2b) compared to vehicle-treated macrophages. Additionally, mature primary macrophages treated with butyrate also significantly upregulated efferocytosis efficiency and capacity compared to vehicle-treated macrophages, regardless of the nature of the AC target (Fig. 2c and Extended Data Fig. 2a). Additionally, the boost in efferocytosis capacity and efficiency provided by butyrate was maintained for up to 3d after withdrawal of butyrate *in vitro* (Fig. 2d and Extended Data Fig. 2b).

Because TRMs are the primary professional phagocyte responsible for clearing millions of ACs daily^1^, the ability to internalize multiple ACs simultaneously is likely an essential ability^20^. We therefore asked whether butyrate treatment of primary macrophages alters efferocytosis capacity on a per-cell basis. To assess this, we performed time-lapse confocal microscopy of vehicle- and butyrate-treated primary macrophages cultured with ACs. The majority of vehicle-treated macrophages (~80%) engulfed one AC, with all vehicle-treated macrophages engulfing one or two ACs (Fig. 2e). Contrarily, a significantly higher fraction of butyrate-treated macrophages (~40%) engulfed two or more ACs, with several macrophages engulfing three or more ACs (Fig. 2e). We found that butyrate-treated macrophages exhibited a pronounced change in morphology, exemplified by the extension of numerous filopodia (Extended Data Fig. 2c). Such filopodial extensions are thought to correspond with a faster form of phagocytosis and to be important for efficient sensing of cell death and phagocytosis by embedded TRMs^21^. We found that the benefit of butyrate extends to human macrophages, as butyrate treatment significantly enhanced efferocytosis by primary human monocyte-derived macrophages (Fig. 2f). Unlike most forms of phagocytosis, homeostatic efferocytosis is anti-inflammatory. Nevertheless, the factors that support different types of phagocytosis remain understudied. Thus, we tested if the effect of butyrate on macrophage efferocytosis extends to other forms of phagocytosis. Surprisingly, treatment with butyrate had no effect on bacterial phagocytosis (either *E. coli* or *S. aureus*; Fig. 2g), latex bead phagocytosis, or fungal particles (Extended Data Fig. 2d) consistent with a previous study that found no effect of butyrate on bacterial phagocytosis^22^. Collectively, our findings suggest that butyrate uniquely informs mouse and human macrophage homeostatic efferocytosis potential.

In order to better understand why macrophages treated with butyrate are more efficient phagocytes, we performed bulk mRNA sequencing (RNA-seq) of primary macrophages treated with butyrate or vehicle control. Analysis of butyrate-treated macrophages revealed differential regulation of numerous cell biological function-associated transcriptional programs (Fig. 2h and Supplementary Table 1). For example, butyrate-treated macrophages significantly upregulate both carbohydrate and lipid metabolism programs but downregulate both oxidative stress/redox biology and ER Stress/Unfolded Protein Response (UPR) programs (Fig. 2h). Broadly, our RNA-seq analysis suggests that butyrate treatment of primary macrophages enforces core TRM programs important for homeostatic functions, such as efferocytosis.

Given our finding that butyrate treatment enhances efferocytosis efficiency and capacity, we next explored in more detail the phagocytosis transcriptional program induced by butyrate treatment. The phagocytosis transcriptional program is a broad categorization that includes various homeostatic- and infection-associated programs. We observed that butyrate treatment significantly downregulated genes associated with bacterial phagocytosis, oxidative stress/redox biology, and pro-inflammatory signaling (Fig. 2i, Extended Data Fig. 2e, and Supplementary Table 1) within the phagocytosis program. Contrarily, we observed significant upregulation of genes associated with efferocytosis (Fig. 2i, Extended Data Fig. 2e, and Supplementary Table 1) within the phagocytosis program. Efferocytosis consists of discrete steps that require distinct cell biological processes and signaling machinery^23^. RNA-seq analysis of butyrate-treated macrophages also revealed significant upregulation of transcriptional programs putatively involved in or supportive of efferocytosis, such as GTPase activity, cell volume regulation, and focal adhesion (Fig. 2j, Extended Data Fig. 2f, and Supplementary Table 1). Taken together, our data suggest that butyrate induces transcriptional programs in macrophages that support core TRM functions, especially homeostatic efferocytosis.

**Fig. 3:**
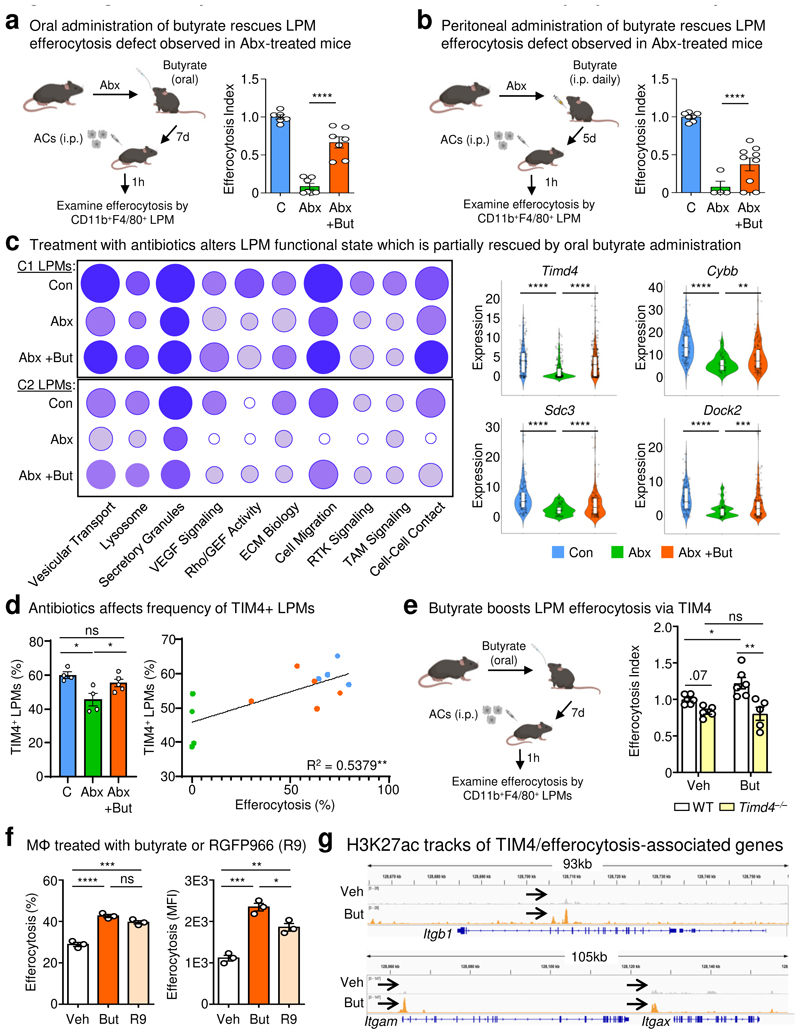
Exogenous butyrate rescues broad-spectrum antibiotic-induced defects in LPM efferocytosis. (a) LPMs efferocytosis efficiency in control mice (n = 6), in mice treated with ABX (ABX, n = 7), and in mice with ABX supplemented with butyrate (150 mM) in drinking water (ABX+But, n = 7). Data are from two independent experiment with three to four mice per experiment. (b) LPMs efferocytosis efficiency in control mice (n = 7), mice treated with ABX (ABX, n = 4), and in mice treated with ABX supplemented with i.p. butyrate (40 mM) (ABX+But, n = 9). Data are from two independent experiments with two to five mice per experiment. (c) Functional programs enriched in clusters C1 and C2 LPMs (left) and representative genes (right) identified by single cell RNA-seq analysis of peritoneal cells from control mice (n = 127), mice treated with ABX (n = 59), and mice treated with ABX and oral butyrate (n = 132). (d) Expression of the efferocytosis receptor TIM4 in efferocytotic LPMs treated as in (a). Percentage of TIM4^+^ LPMs (left) and XY plot showing the linear correlation between the percentage of TIM4^+^ LPMs and efferocytosis capacity (right). (e) LPMs efferocytosis efficiency from wildtype (WT) or TIM4-deficient (*Timd4*^–/–^) mice supplemented with oral butyrate (150 mM). Untreated wildtype (n = 6), untreated TIM4-deficient (n = 5), butyrate-treated wildtype (n = 6), and butyrate-treated TIM4-deficient (n = 5). Data are from two independent experiment with two to three mice per experiment. (f) Efferocytosis efficiency (left) and capacity (right) by mature primary macrophages conditioned with vehicle, butyrate (1 mM), or RGFP966 (R9; 20 µM) for 3d. Data are from three independent experiments. (g) Chromatin immunoprecipitation sequencing (CHIPseq) of H3K27 acetylation (H3K27ac) DNA binding sites. Comparison of H3K27ac CUT&RUN tracks generated from macrophages treated with butyrate (orange) or a vehicle control (grey). Comparisons for three significantly differential peaks associated with TIM4/efferocytosis related genes are shown. Data are from three independent experiments. All bar graphs represent means ± s.e.m. Statistics were performed by one-way ANOVA in **a, b, d**, and **f**, two-way ANOVA in **e**. *p < .05; **p < .01; ***p < .001. ****p < .0001. ns - not significant. Schematics were created with BioRender.com.

We next sought to determine if butyrate availability could account for the defective homeostatic efferocytosis observed in ABX-treated mice. To test this, we took two independent *in vivo* approaches. First, we supplemented the drinking water of ABX-treated mice with butyrate to imitate the conventional route of access (Fig. 3a). Similar to our previous observations, homeostatic efferocytosis was significantly decreased in ABX-treated mice compared to untreated mice (Fig. 3a). On the other hand, ABX-treated mice orally supplemented with butyrate exhibited significantly rescued homeostatic efferocytosis capacity (Fig. 3a). Second, we delivered butyrate directly to the peritoneal cavity, allowing us to test both the direct relevance of butyrate on LPM efferocytosis *in vivo* and to bypass potential confounds that might arise from dysbiosis in the intestines (Fig. 3b). Similar to oral supplementation, peritoneal delivery of butyrate significantly rescued LPM efferocytosis in ABX-treated mice (Fig. 3b). Thus, intestinal microbiome-derived and locally-delivered butyrate supports homeostatic efferocytosis by LPMs.

**Extended Data Fig. 3:**
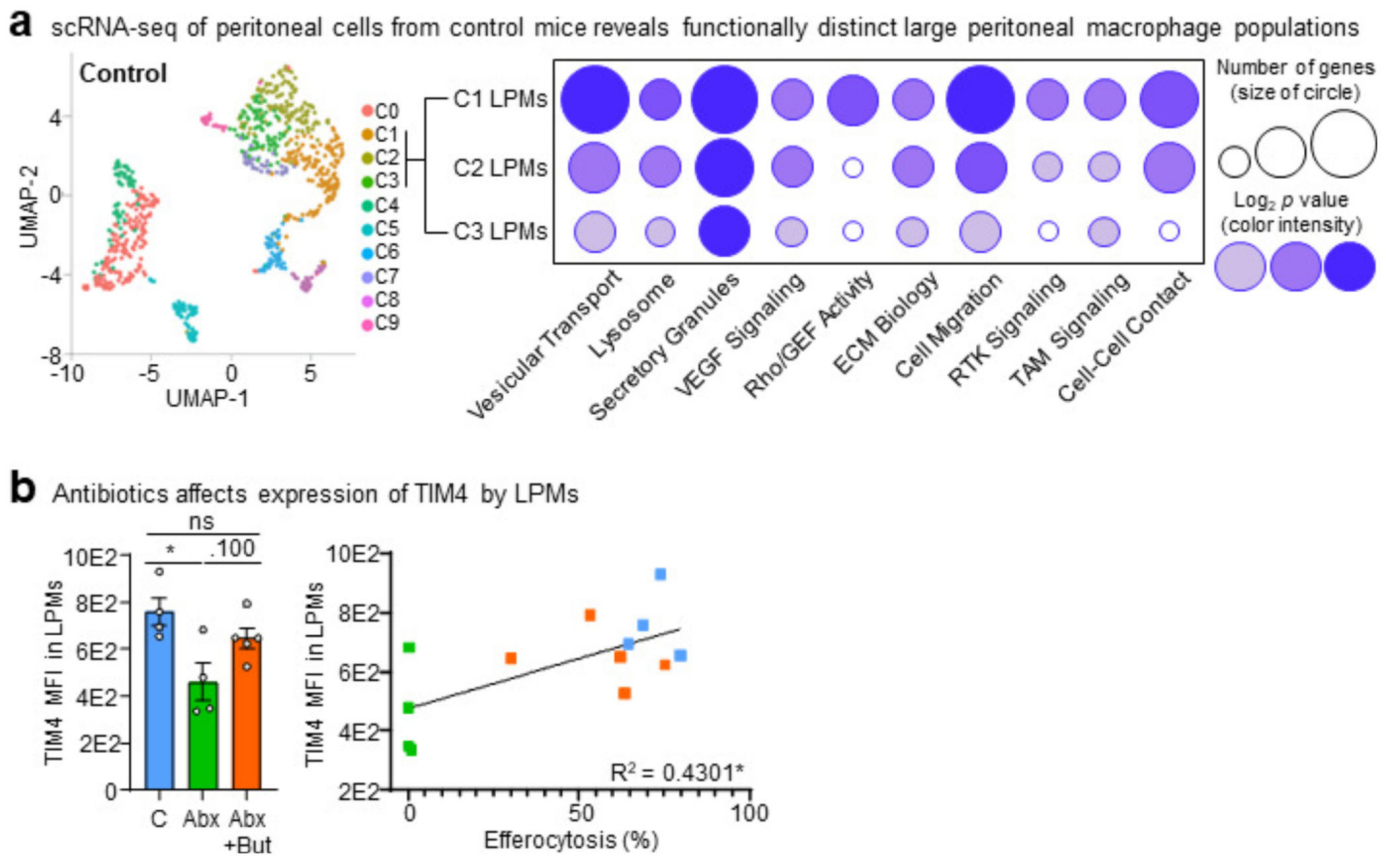


**Extended Data Fig. 4:**
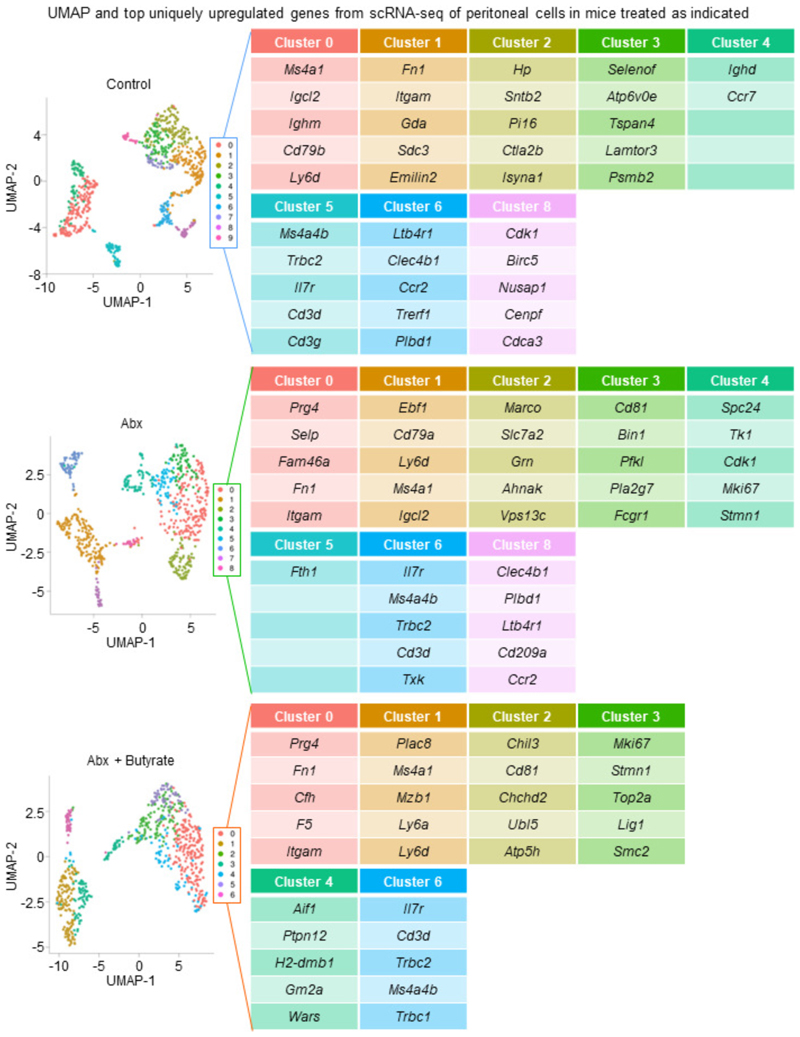


**Extended Data Fig. 5:**
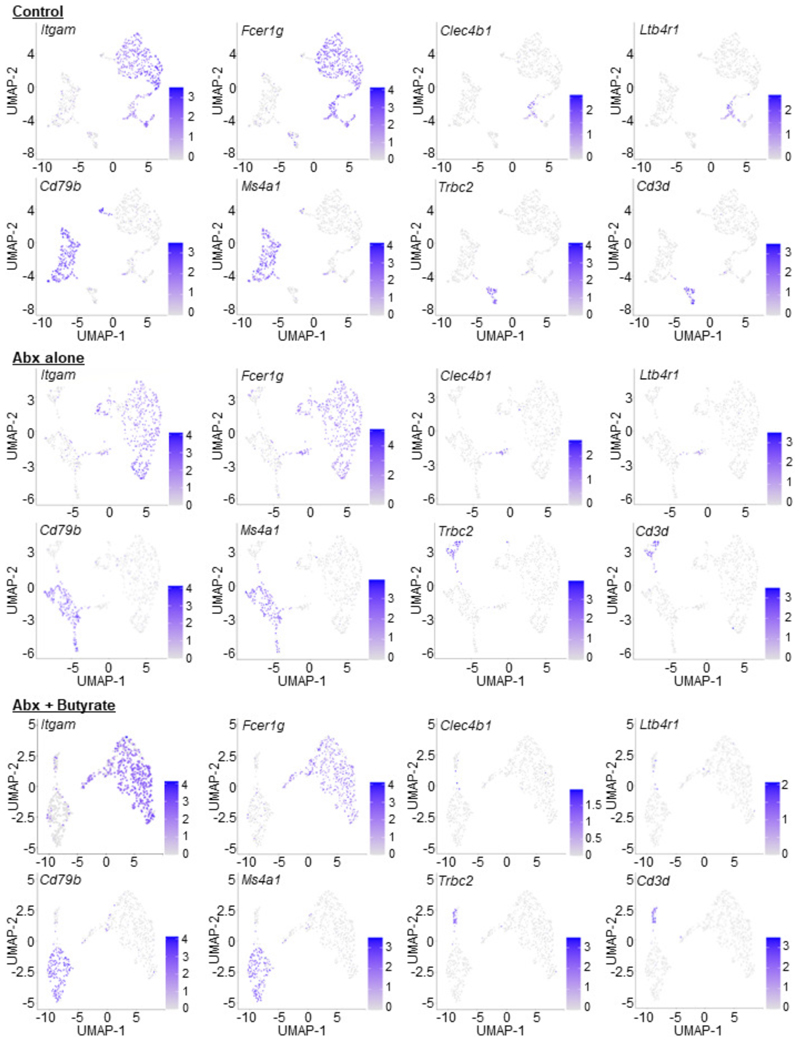


Although we could rescue LPM efferocytosis by providing butyrate locally, it remained possible that microbiota-derived butyrate does not act directly on LPMs. To further test this hypothesis, we performed single cell RNAseq (scRNA-seq) of peritoneal immune cells isolated from control mice, ABX-treated mice, and ABX-treated mice supplemented with oral butyrate (Fig. 3c and Extended Data Fig. 3a). We initially performed an unbiased analysis of the peritoneal immune cell compartment to determine if ABX treatment affected the peritoneum more broadly. However, the combination of immune cell type identification via cell type-specific transcription profiles curated by the Immunological Genomes project^24^ together with uniform manifold approximation and projection (UMAP) analysis revealed a consistent presence of key immune cell subsets^25^, including B1 B Cells (*Ms4a1*+), T cells (*Il7r*+), Small Peritoneal Macrophages (SPMs; *Clec4b1*+), and LPMs (*Itgam*+, *Adgre1*+), across all three conditions (Extended Data Fig. 4 and 5). Furthermore, based on Immunological Genome project data^24^, we identified four overlapping but distinct LPM populations in untreated mice, characterized by differential transcriptional expression of F4/80 (*Adgre1*), CD11b (*Itgam*), FCER1G (*Fcer1g*), and LysM (*Lyz2*). The predominant LPM population in untreated mice (Cluster 1) most closely resembled the canonical phagocytic peritoneal TRM, featuring robust expression of *Adgre1* and *Itgam* as well as significant expression of efferocytosis receptors *Timd4, Mertk*, and *Tyrobp* (Extended Data Fig. 3 and 4; Supplementary Table 2-4). This population also accounted for the majority of individual LPMs. The second LPM population (Cluster 2) also featured significant *Adgre1* and *Itgam* expression as well as increased *Timd4* and *Tyrobp* expression but did not exhibit enhanced *Mertk* expression (Extended Data Fig. 3 and 4; Supplementary Table 2-4). The third (Cluster 3) and fourth (Cluster 7) LPM populations featured enrichment of genes uniquely found in LPM RNA-seq from ImmGen but were instead predominantly characterized by enrichment of other previously identified LPM genes, such as *Fcer1g* (Cluster 3) and *Lyz2* (Cluster 7) and lacked the canonical efferocytosis receptors *Timd4* and *Mertk* (Extended Data Fig. 3 and 4; Supplementary Table 2-4). Nonetheless, identification of LPM clusters allowed us to perform downstream analysis of the impact of ABX treatment and butyrate rescue on LPM functional programs.

In depth analysis of LPMs from control mice revealed Cluster 1 and Cluster 2 LPMs were uniquely enriched for functional programs putatively involved in or supportive of efferocytosis (Fig. 3c and Supplementary Table 5-8). Specifically, we observed enrichment of programs, including putative efferocytosis programs, also observed in macrophages treated with butyrate *in vitro*, including vesicular transport, lysosome biology, Rho/GEF activity, ECM biology, cell migration, receptor tyrosine kinase (RTK) signaling, and cell-cell contact/cell adhesion (Fig. 3c and Supplementary Table 5-8). Both Cluster 1 and Cluster2 LPMs from ABX-treated mice exhibited significant decreases across analyzed efferocytosis-associated functional programs, especially lysosome biology, Rho/GEF activity, and RTK signaling (Fig. 3c). Furthermore, amongst identified Cluster 1 and Cluster 2 LPMs in ABX-treated mice, there were fewer cells expressing core efferocytosis transcripts and the expression level of core transcripts was lower (e.g., *Timd4*; Fig. 3c and Supplementary Table 5-8). However, both Cluster 1 and Cluster 2 LPMs from ABX-treated mice receiving oral butyrate supplementation exhibited a significant rescue of efferocytosis-associated functional programs (Fig. 3c).

Our RNA-seq analyses of butyrate-treated primary macrophages *in vitro* and ABX-treated LPMs *in vivo* suggested that butyrate supports homeostatic efferocytosis by directly regulating expression of efferocytosis-associated transcripts. We were particularly drawn to the efferocytosis receptor, T-cell immunoglobulin and mucin domain containing 4 (TIM-4, *Timd4*), because of the following findings: 1) *Timd4* was upregulated in butyrate-treated macrophages *in vitro* (Fig. 2h,i and Supplementary Table 1), 2) *Timd4* was downregulated in LPMs and fewer LPMs expressed *Timd4* in ABX-treated mice (Fig. 3c and Supplementary Table 5-8), and 3) both the level of *Timd4* expression in LPMs and the numbers of *Timd4*-expressing LPMs was rescued in ABX-treated mice supplemented with butyrate (Fig. 3c and Supplementary Table 5-8). Thus, we hypothesized that TIM-4 expression, at least in part, underlies the decreased LPM efferocytosis capacity observed in ABX-treated mice. To test this, we first analyzed the protein expression of TIM-4 by LPMs in both ABX-treated mice and ABX-treated mice supplemented with oral butyrate. We observed both significantly lower frequency of TIM-4-expressing LPMs and decreased TIM-4 expression by LPMs in ABX-treated mice (Fig. 3d and Extended Data Fig. 3b). On the other hand, we found that butyrate supplementation significantly rescued both the frequency of TIM-4-expressing LPMs and TIM-4 expression by LPMs in ABX-treated mice (Fig. 3d and Extended Data Fig. 3b). Both the frequency of TIM-4-expressing LPMs and TIM-4 expression by LPMs significantly correlated with LPM efferocytosis capacity (Fig. 3d and Extended Data Fig. 3b). Finally, we found that TIM-4-deficiency completely reverses the butyrate-induced boost of homeostatic efferocytosis by LPMs *in vivo* (Fig. 3e). Thus, our data suggest that microbiome-derived butyrate supports efficient homeostatic efferocytosis by LPMs through direct regulation of efferocytosis-associated proteins, including the efferocytosis receptor TIM-4.

**Extended Data Fig. 6:**
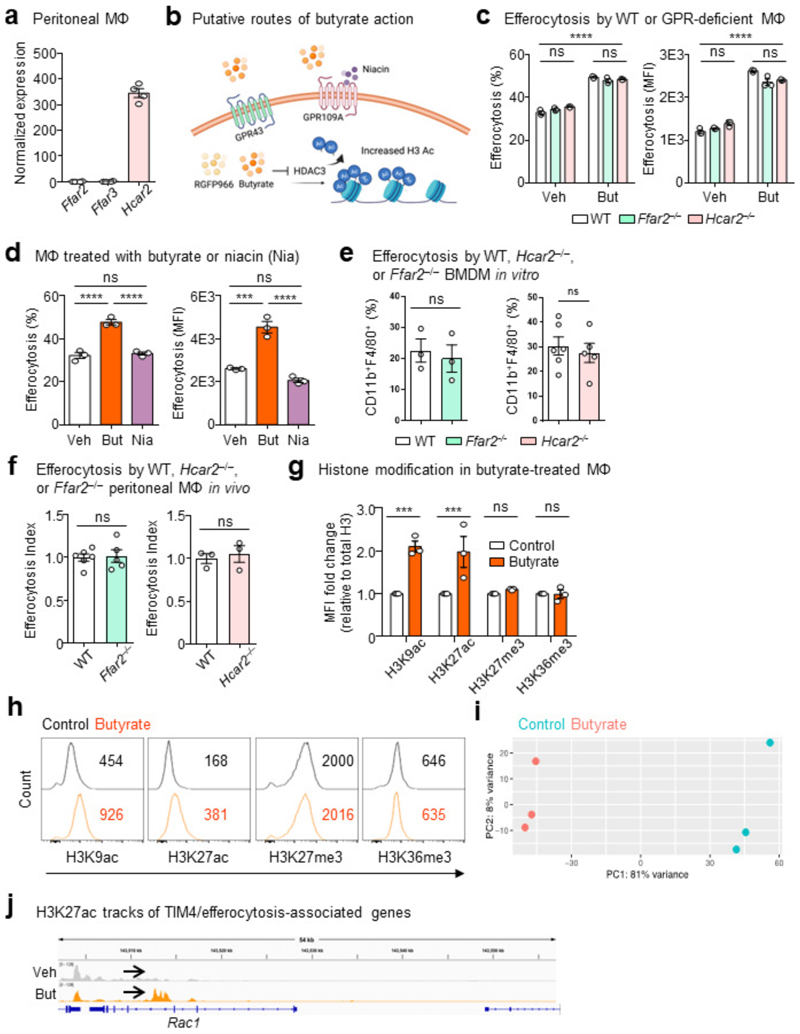


RNA-seq analysis revealed that LPMs, at least transcriptionally, appear to express the cognate butyrate GPCR GPR109a (*Hcar2*), with negligible expression of the other known SCFA GPCRs GPR41 (*Ffar3*) or GPR43 (*FFar2*) (Extended Data Fig. 6a), consistent with publicly available data from BioGPS and ImmGen databases^24,26^. Accordingly, we first investigated whether GPCR signaling could account for the enhanced efferocytosis promoted by butyrate (Extended Data Fig. 6b). Contrary to our hypothesis, macrophages lacking GPR109a exhibited enhanced efferocytosis capacity in response to butyrate *in vitro* (Extended Data Fig. 6c). Given previous studies have reported macrophage function relying on GPR43^18^, we also tested whether GPR43 is required for butyrate-mediated enhanced efferocytosis. Similar to our results with GPR109a-deficient macrophages, GPR43-deficient macrophages treated with butyrate exhibited significantly enhanced efferocytosis capacity *in vitro* (Extended Data Fig. 6c). Additionally, culturing macrophages with the GPR109a agonist niacin failed to enhance efferocytosis capacity (Extended Data Fig. 6d). To further assess if GPR109a or GPR43 are required for homeostatic efferocytosis by TRMs, we performed homeostatic efferocytosis assays in the peritoneum of GPR109a- and GPR43-deficient mice. Consistent with our *in vitro* results, we observed no significant differences in LPM homeostatic efferocytosis capacity or LPM frequency between wildtype and GPR109a-deficient mice or between wildtype and GPR43-deficient mice (Extended Data Fig. 6e,f). Taken together, our results suggest that butyrate signaling via known cognate GPCRs is dispensable for efficient homeostatic efferocytosis.

We next investigated whether direct HDAC inhibition impacts efferocytosis capacity. To this end, we treated macrophages with RGFP966, a specific inhibitor of the known butyrate target HDAC3 (Extended Data Fig. 6b)^22^. Inhibition of HDAC3 significantly enhanced efferocytosis efficiency and capacity to a level similar to butyrate-conditioned macrophages (Fig. 3f), suggesting that butyrate supports homeostatic efferocytosis through inhibition of HDAC3.

Additionally, treatment with butyrate increased the acetylation of both lysines 9 and 27 on histone 3 (H3K9ac and H3K27ac) but had no effect on tri-methylation of lysines 27 and 36 (H3K27me3 and H3K36me3) (Extended Data Fig. 6g,h), further supporting the notion that butyrate supports homeostatic efferocytosis via cell-intrinsic transcription regulation via histone deacetylation. Chromatin immunoprecipitation sequencing (CHIPseq) revealed significant butyrate-induced binding of acetylated H3K27 to genes responsible for TIM4-mediated efferocytosis, including *Itgb1, Itgam, Itgax*, and *Rac1* (Fig. 3g, Extended Data Fig. 6i,j, and Supplementary Table 9). Our results suggest that microbiome-derived butyrate functions to transcriptionally ‘prepare’ tissue-resident macrophages for homeostatic functions such as efferocytosis.

**Extended Data Fig. 7:**
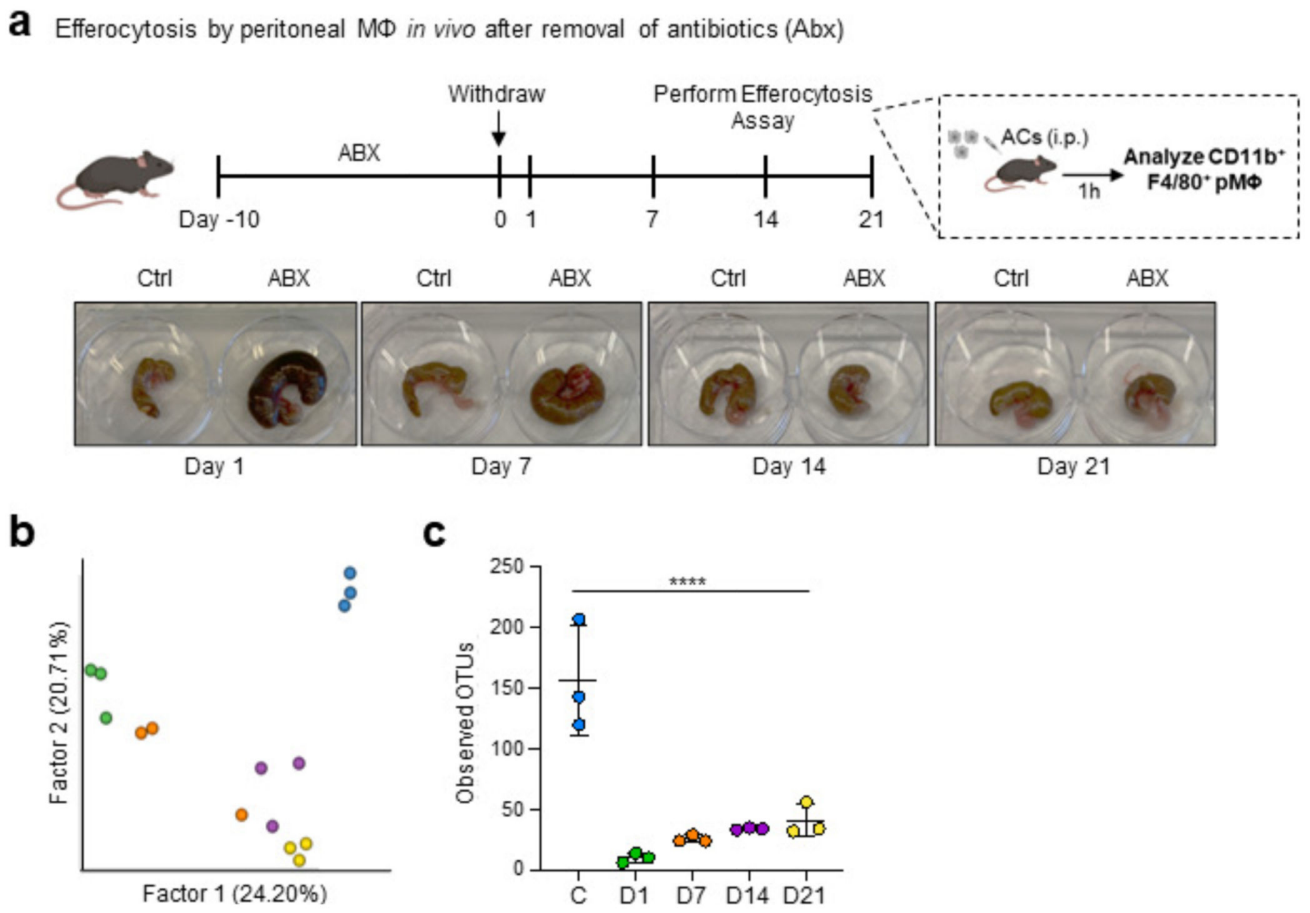


**Fig. 4:**
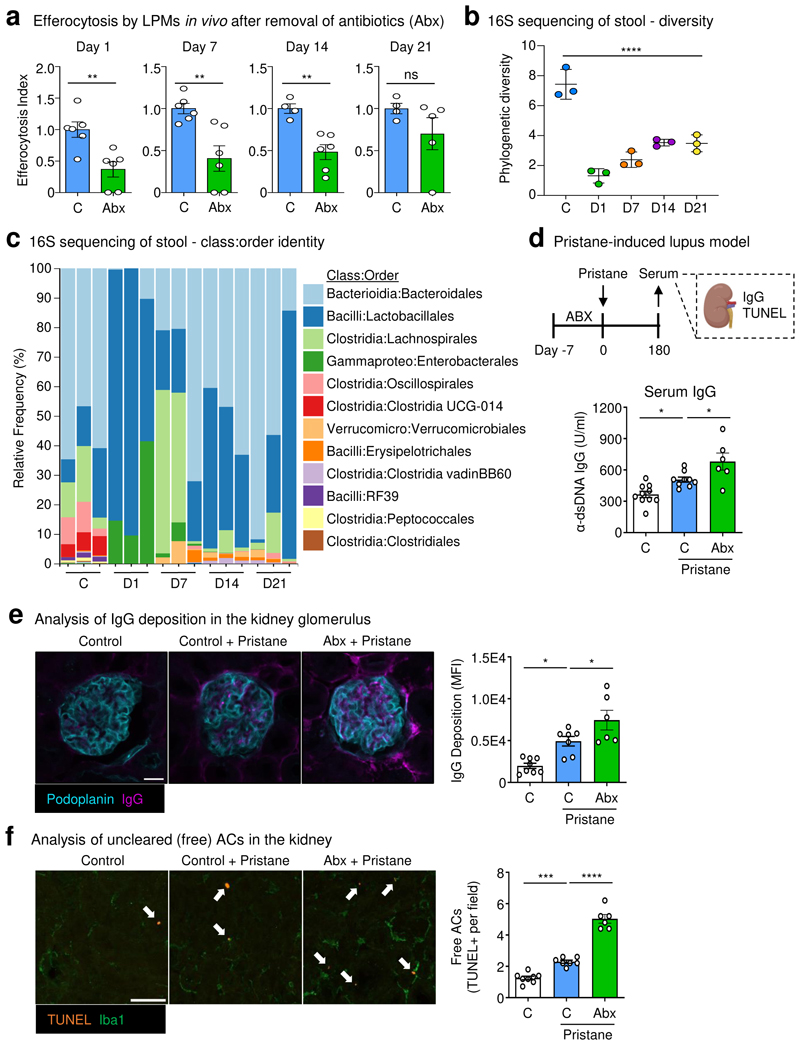
Treatment with antibiotics induces prolonged peripheral efferocytosis defects. (a) Mice were treated with broad-spectrum antibiotics (ABX) in drinking water for 10d then back on normal drinking water. On indicated days post-ABX withdrawal, LPMs were analyzed for efferocytosis efficiency. Data are from day 1 (control n = 6, ABX n = 6), day 7 (control n = 6, ABX = 6), day 14 (control n = 4, ABX = 6), and day 21 (control = 4, ABX = 5) post-ABX withdrawal across two independent experiments. (b, c) 16S sequencing analysis of mouse stool from experiments performed in (a). Shown is the Faith’s phylogenetic (alpha) diversity (n = 3) (b) and the relative frequency of key bacterial orders (c). Data are from three biological replicates. (d-f) Control or ABX-treated mice were injected with a single dose of pristane (500 µL per mouse) and monitored. On day 180 post-pristane injection, serum and tissues were collected and analyzed. (d) α-dsDNA IgG quantification in the serum of untreated mice (n = 10), control & pristane-treated mice (n = 9), and ABX-treated & pristane-treated mice (n = 6). (e) Representative immunofluorescence (IF) images (left) and summary plot of the analysis of α-dsDNA IgG deposition in the kidney glomerulus of untreated mice (n = 8), control & pristane-treated mice (n = 7), and ABX-treated & pristane-treated mice (n = 6). (f) Representative IF images (left) and summary plot of the analysis of apoptotic cells using terminal deoxynucleotidyl transferase biotin-dUTP nick end labeling (TUNEL) in the kidney of untreated mice (n = 8), control & pristane-treated mice (n = 7), and ABX-treated & pristane-treated mice (n = 6). White arrows indicate uncleared apoptotic cells (TUNEL+ events), e.g., TUNEL+ events not touching Iba1+ macrophages. Scale bars are 20 µm (e) and 50 µm (f). All bar graphs represent means ± s.e.m. Statistics were performed by two-tailed *t-*test in **a**, one-way ANOVA in **b, d, e** and **f**. *p < .05; **p < .01; ***p < .001. ****p < .0001. ns - not significant. Schematics were created with BioRender.com.

We next queried whether treatment with ABX induced disruption of homeostatic efferocytosis beyond the end of the ABX course (Extended Data Fig. 7a). Consistent with our previous experiments, we observed decreased homeostatic efferocytosis by LPMs in ABX-treated mice one day post-ABX withdrawal (Fig. 4a and Extended Data Fig. 7a). This deficiency corresponded with the hallmark enlarged, dark-colored cecum found in mice with dysbiosis (Extended Data Fig. 7a). Homeostatic efferocytosis by LPMs remained significantly impaired beyond two weeks post-ABX withdrawal, despite the apparent reversal cecum gross pathology (Extended Data Fig. 7a). We also performed analysis of mouse stool via 16S sequencing to further detail the specifics of ABX-induced dysbiosis and recovery. As expected, we observed dramatic loss of microbiome diversity in ABX-treated mice when assessed one day after ABX withdrawal (Fig. 4b and Extended Data Fig. 7b,c). ABX treatment caused a significant loss of multiple known butyrate-producing *Clostridia* species (Fig. 4c). Furthermore, microbiome diversity remained significantly decreased throughout the course of the experiment, with only modestly increased microbial diversity 14 and 21 days post-ABX removal (Fig. 4b and Extended Data Fig. 7b,c), which corresponds with the slow recovery of homeostatic efferocytosis capacity in LPMs (Fig. 4a). Thus, treatment with broad-spectrum antibiotics results in prolonged dysbiosis and concomitant homeostatic efferocytosis deficiency.

Lastly, we sought to determine whether treatment with ABX affects the severity of an efferocytosis-dependent autoimmune disease model. To this end, we used the pristane model of systemic lupus erythematosus (SLE) which, similar to human SLE, depends on apoptosis of cells in both lymphoid organs and the kidney for disease induction and progression^27^. Specifically, mice were treated with a 7-day course of ABX-containing water or normal water prior to pristane injection (Fig. 4d). Mice treated with ABX exhibited significantly worse lupus-like disease compared to untreated mice as defined by increased anti-double-stranded DNA (α-dsDNA) immunoglobulin G (IgG) antibodies in the serum (Fig. 4d) and increased deposition in the kidney glomerulus (Fig. 4e). Additionally, we observed that ABX-treated mice displayed significantly more uncleared apoptotic cells in the kidney (Fig. 4f), consistent with the observation that cell death in the kidney precipitates lupus pathogenesis. Collectively, our new data directly supports the hypothesis that broad-spectrum antibiotics perturbs homeostatic efferocytosis and may lead to increased susceptibility to autoimmunity.

The ability of phagocytes to clear apoptotic cells is a key facet of tissue health and organismal homeostasis. Indeed, organisms that exhibit perturbations of homeostatic efferocytosis machinery often present with inflammatory sequalae which, in mammals, can result in the development of autoimmunity or inflammatory disease^2,3^. How this ability is established and maintained in tissue-resident phagocytes (such as tissue-resident macrophages, TRMs) and whether signals arising from distal sites (such as the intestinal tract) contribute remains poorly understood. Here, across multiple lines of investigation, we made the surprising discovery that TRM’s ability to perform homeostatic efferocytosis in the peritoneum is informed by signals arising from the intestinal microbiome. Specifically, we found that large peritoneal macrophages (LPMs) in mice treated with ABX or vancomycin exhibit significant homeostatic efferocytosis defects. This defect was attributable to the absence of the intestinal microbiome, as (1) germ-free mice also exhibited a significant decrease in homeostatic efferocytosis, and (2) ABX treatment-induced homeostatic efferocytosis defects were rescued via fecal microbiota transplantation. Mechanistically, we discovered that the short-chain fatty acid butyrate is an essential distal signal capable of rescuing homeostatic efferocytosis defects caused by ABX treatment. Through a combination of cell biological, bulk and single cell RNA sequencing, and *in vivo* animal approaches, we show that butyrate acts directly on LPMs to support homeostatic efferocytosis. Specifically, mouse and human macrophages conditioned with butyrate exhibited enhanced efferocytosis efficiency and capacity whereas butyrate had no effect on other forms of inflammatory phagocytosis, including bacterial or fungal phagocytosis. The effect of butyrate on homeostatic efferocytosis was accomplished through upregulation of the efferocytosis receptor TIM-4, as ABX treatment resulted in both fewer TIM-4-expressing LPMs and decreased expression of TIM-4 in LPMs, whereas TIM-4-deficient mice lacked the butyrate-induced increase in homeostatic efferocytosis. Additionally, both oral and intraperitoneal administration of butyrate rescued the homeostatic efferocytosis defect observed in LPMs of ABX-treated mice *in vivo*. We observed that the defect in homeostatic efferocytosis persists beyond ABX withdrawal and that mice treated with ABX exhibited significantly worse disease in the pristane-induced mouse model of system lupus erythematosus. Our work provides an additional possible link between the clinical observation that patients receiving ABX are susceptible to the development of inflammatory disease and autoimmunity in non-intestinal tissues^4–7,28^. Taken together, we propose that TRMs rely on integration of distal molecular signals to establish and maintain their ability to perform homeostatic efferocytosis.

The importance of SCFAs, especially butyrate, on host immune cell function in the intestines is well-established^18^, though its importance for macrophage function is less straightforward. A recent study suggested that butyrate, despite suppressing inflammatory cytokine production, programs monocyte-derived macrophages into effective antimicrobial macrophages including increased production of antimicrobial products in response to bacterial infection *in vitro* and in the intestines *in vivo*^22^. However, this study found that butyrate had no effect on bacterial phagocytosis consistent with our own findings. To our knowledge, our work is the first to examine the role of SCFAs, in particular butyrate, on non-inflammatory phagocytosis (homeostatic efferocytosis). Our work and that of previous groups suggests that SCFAs and other bacterial metabolic byproducts differentially regulate TRM function depending on tissue context (e.g., inflammatory versus homeostatic), begging the question of whether butyrate or other SCFAs function differently during homeostatic and inflammation-resolving efferocytosis.

Our work raises several additional important questions. For instance, we observed that exogenous butyrate, regardless of the route of supplementation, resulted in an approximate ˜50% rescue of efferocytosis by LPMs. What additional intestinal microbiome-derived signals support homeostatic efferocytosis by LPMs? Although we report that efferocytosis is impaired in ABX-treated mice in the peritoneum, thymus, and kidney, are other TRM-specific clearance processes, such as the removal of mitochondria in the heart^29^ or surfactant in the lung^30^, also affected? Relatedly, most tissues have both professional and non-professional phagocytes that perform unique tissue-specific clearance activities^3^. Do distal signals affect professional and non-professional phagocytes differently? Finally, do host tissues generate molecular signals that act distally to inform homeostatic efferocytosis in other tissues? Nevertheless, the current study provides a roadmap to further our understanding of how TRMs are programmed to perform homeostatic efferocytosis and maintain tissue homeostasis. The data presented here advance the concept that metabolites produced by commensal microbes act distally to enforce efficient efferocytosis by TRMs and may help to explain the epidemiological observation that patients treated with ABX exhibit increased incidence of autoimmune and autoinflammatory diseases.

## Methods

### Contact for Reagent and Resource Sharing

Requests for resources and reagents should be directed to the Lead Contact, Justin S. A. Perry (perryj@mskcc.org).

### Animal studies

Wild-type C57BL/6J (Stock No. 000664) and J:DO (Stock No. 009376) were purchased from The Jackson Laboratory. *Ffar2*^*-/-*^ and *Hcar2*^*-/-*^ mice (C57BL/6J background) were provided by M.v.d. Brink. *Timd4*^*-/-*^ mice (C57BL/6J background) were provided by T. Merghoub and J. Wolchok. Germ-free mice were provided by A. Rudensky, maintained in flexible isolators, and routinely checked for bacteria and fungi by PCR of fecal DNA samples for bacterial 16S and fungal 18S, respectively. All mice were housed at the Research Animal Resource Center for MSKCC (specific-pathogen free) and Weill Cornell Medicine (germ-free) with 12-hour light/dark cycles. Mice were housed in ambient conditions and *ad libitum* access to water and food (LabDiet, 5053). For all experiments, mice (male and female) were used at 8-12 weeks of age. All studies were conducted under protocol number 19-07-012 and approved by the Sloan Kettering Institute (SKI) Institutional Animal Care and Use Committee (IACUC). Animals were randomly assigned to experimental conditions, and investigators were not blind to the conditions of the experiments.

### Reagents

The reagents used in this work were as indicated: CypHer5E (GE Life Sciences, PA15401); CellTrace Yellow (Thermo Fisher, C34567); Hoechst (Thermo Fisher, H3570); sodium butyrate (Sigma, 303410, 1 mM); sodium acetate (Sigma, S2889, 1 mM); sodium propionate (Sigma, P1880, 1 mM); RGFP966 (Selleck Chemicals, S7229, 20 µM); niacin (Sigma, N4126, 500 µM).

### Bone marrow-derived macrophages

BMDMs were generated by culturing mouse bone marrow cells in alpha-MEM (Fisher Scientific, MT15012CV) containing 10% (vol/vol) heat-inactivated FBS (Sigma), 10% (vol/vol) L929 cell (ATCCCCL-1)-conditioned medium, 100 U/ml penicillin, 100 µg/ml streptomycin, and L-glutamine 37°C and 5% CO_2_ for 7 days. BMDMs were seeded at indicated densities and allowed to rest overnight prior experiments.

### Human monocyte-derived macrophages

Peripheral blood mononuclear cells (PBMC) derived from anonymous healthy blood donors (New York Blood Center) were obtained by gradient separation using 1-Step Polymorphs (Accurate Chemical, AN221725). CD14^+^ monocytes were isolated with the Mojosort human CD14 nanobeads (BioLegend, 480093). 5x10^6^ monocytes were seeded and differentiated into macrophages for 5 days in RPMI-1640 (Corning, 17-105-CV) supplemented with 10% (vol/vol) heat-inactivated FBS, 100 U/ml penicillin, 100 µg/ml streptomycin, L-glutamine, and 100 ng/ml recombinant human M-CSF (PeproTech, 300-25) at 37 °C and 5% CO_2_. HMDMs were seeded at indicated densities and allowed to rest overnight prior experiments.

### Antibiotic treatment

Mice were given a cocktail of antibiotics or individual antibiotic in drinking water for at least 7 days prior experiments, unless otherwise stated. Antibiotic cocktail consisted of 1 g/L ampicillin (Cayman Chemical, 14417); 1 g/L kanamycin (K4000); 0.5 g/L metronidazole (Sigma, M3761); 0.5 g/L vancomycin (BioVision, B1507).

### Fecal microbiota transplant

About 6-8 stool pellets from SPF mice were collected into sterile micro tubes containing micro beads (Sarsted, 72.693.005), resuspended in sterile PBS, and vortexed until stool pellets were dissolved in solution. Fecal homogenates were centrifuged at max speed, and 200 µL were administered to mice through oral gavage. Mice were used for experiments 7 days after microbiota reconstitution.

### In vitro efferocytosis and phagocytosis

Apoptosis was induced in mouse primary thymocytes or human Jurkat T cells (ATCC TIB-152) cultured in RPMI supplemented with 5% FBS by 150 mJ/cm ultraviolet C irradiation. Thymocytes or Jurkat cells were incubated at 37 °C and 5% CO_2_ for 4 hours, followed by staining with the pH sensitive dye CypHer5E or CellTrace Yellow according to the manufacturer’s instructions before efferocytosis assay. After staining, apoptotic cells were resuspended in BMDM media. 1 x 10^5^ BMDMs were seeded in 24-well plates and stained with 2 µg/ml Hoechst to discriminate phagocyte from target. BMDMs were incubated with target cells at a 1:1 (Jurkat cells) or 1:10 (thymocytes) phagocyte:target ratio for the indicated times. For alternative types of phagocytosis, Hoechst-stained BMDMs were incubated with Alexa Fluor 594-conjugated zymosan A (Z23374; Thermo Scientific), *E. coli* (E23370; Thermo Scientific), *S. aureus* (S23372; Thermo Scientific) or latex beads (L3030; Sigma). Cells were scrapped off wells and phagocytes (Hoechst^+^) were assayed on an Attune NxT cytometer (Thermo Scientific). Samples were analyzed with FlowJo v10.8.1 (BD). Transluminescece images were acquired with EVOS M5000 software v1.4. For live-cell imaging, Hoechst-stained BMDMs were incubated with CypHer5E-stained apoptotic Jurkats, and imaged every 5 minutes on a Zeiss LSM 980 with Airyscan 2 (20X objective) and acquired with Zen Blue v3.0.

### In vivo efferocytosis

Mice were intraperitoneally injected with 1 x 10^6^ CypHer5E-labelled apoptotic Jurkat cells and euthanized 1-hour post-injection. Peritoneal lavage was collected in 8 ml cold PBS. Collected lavage was spun down and cells were blocked with CD16/32 anti prior being stained with CD11b eFluor 450 (eBioscience, 48-0112-82, clone M1/70, 1:200), F4/80 PE (eBioscience, 12-4801-82, clone BM8, 1:400), and TIM-4 PE-Cy7 (BioLegend, 130009, clone RMT4-54, 1:400) for 30 min at 4 °C. Efferocytosis was analyzed by measuring CypHer5E^+^ events within the population of CD11b^+^F4/80^hi^ peritoneal macrophages in the flow cytometer. In separate *in vivo* efferocytosis experiments, six- to eight-week-old mice were injected i.p. with 300μl PBS containing 250 μg dexamethasone (50 μM; Sigma). 6h post-injection, thymi were harvested from mice and the numbers of annexin V/7-AAD double positive cells (secondarily necrotic) were assessed by FACS. The efferocytosis index is calculated by first taking the designated control group, for instance untreated mice, and calculating the average efferocytosis percentage within those animals. We then calculate the variability within control mice by dividing each control mouse replicate by the control group average. We then perform a similar set of calculations, taking each treatment condition mouse and dividing it by the control group average. This series of calculations is performed for each individual experiment, normalizing the data in a way that allows for it to be combined and analyzed statistically.

### Pristane-induced lupus model

Mice were intraperitoneally injected with 500 µL of pristane (P2870; Sigma) 7 days after antibiotics treatment. Serum was collected on indicated time points for quantification of anti-dsDNA antibodies with the Mouse Anti-dsDNA Igs (Total A+G+M) ELISA Kit (5110; Alpha Diagnostics International). Mice were sacrificed 6 months after pristane injection, and kidneys collected for histological analysis.

### Immunofluorescence and confocal microscopy

Mice were euthanized with CO_2_, perfused with 10 mL of cold PBS, and kidneys were fixed in 4% PFA (StatLab; 28530-1) for 24-48 hours at 4°C. Tissues were then washed 3x in PBS, and submerged in 30% sucrose (S0389; Sigma) for 24 hours, followed by embedding and freezing in Tissue-Tek® O.C.T. Compound (4583; Sakura Finetek USA, Inc.). Cryopreserved tissues were sectioned with a cryostat (Leica) into 12 µm sections. After rehydration, sections were blocked with 5% BSA (A7888; Sigma) in PBS for 1 hour at room temperature. For total IgG staining, sections were incubated with goat anti-mouse IgG (H+L) Alexa Fluor 488 (A-11001; Thermo Scientific; 1:500), and Syrian hamster anti-mouse podoplanin (127401, clone 8.1.1; BioLegend; 1:100) for 16 hours at 4°C followed by incubation with goat anti-Syrian hamster Alexa Fluor 647 (107-605-142; Jackson ImmunoResearch; 1:500) secondary antibody for 1 hour at room temperature. For TUNEL staining, sections were stained with the In Situ Cell Death Detection Kit, TMR red according to manufacturer’s instruction (12156792910; Roche), and rabbit anti-Iba1 (019-19741; Wako; 1:400) for 16 hours at 4°C followed by incubation with goat anti-rabbit Alexa Fluor 488 (A-11034; Thermo Scientific; 1:500) secondary antibody for 1 hour at room temperature. Images of kidney sections were taken as Z-stacks on a Zeiss LSM 980 with Airyscan 2 (20X objective) and acquired with Zen Blue v3.0 software. For each mouse, a minimum of 5 fields of view (FOV) were obtained before maximum intensity z projections were created in Fiji for automated quantification of mean fluorescence intensity (MFI) or TUNEL puncta.

### Butyrate treatment

BMDMs were treated with butyrate (1 mM) during or after differentiation for specific experiments. For *in vivo* local treatment, mice were treated intraperitoneally daily with 40 mM butyrate for the indicated times prior experiments. For oral treatment, mice that have been on antibiotic treatment were placed on drinking water with antibiotics supplemented with 100 mM butyrate for 7 days prior experiments.

### Histone acetylation and methylation analysis

For histone analysis, BMDMs were scrapped off wells after indicated treatments. Cells were fixed and permeabilized with the Foxp3 transcription factor staining kit (eBioscience, 00-5523-00) prior intracellular staining with the indicated antibodies: H3K9ac (Cell Signaling, 9649, clone C5B11, 1:3000), H3K27ac (Cell Signaling, 8173, clone D5E4, 1:2000), H3K27me3 (Cell Signaling, 9733, clone C36B11, 1:2500), H3K36me3 (Active Motif, 61021, clone MABI 0333, 1:2000), and total H3 (Active Motif, 39763, clone MABI 0301, 1:500). Samples were then stained with anti-mouse (Invitrogen, A32728, 1:500) or anti-rabbit (Invitrogen, A32733, 1:500) Alexa Fluor 647 secondary antibodies, followed by flow cytometry analysis. Histone acetylation and methylation MFI was normalized to total H3 MFI from matching samples.

### CUT&RUN

CUT&RUN was performed using the EpiCypher CUTANTA ChIC/CUT&RUN kit according to the manufacturer’s protocol. 5x10^6 cells per replicate were harvested for untreated and butyrate treated bone marrow derived macrophages, washed with 1X PBS, and resuspended in wash buffer. After an incubation for 10 minutes with activated Concanavalin A beads, cells were permeabilized and incubated overnight with rabbit IgG (Antibodies Online ABIN101961), or H3K27ac (Cell Signaling Technology 8173S). Cells were then washed with permeabilization buffer and incubated with pA/G-MNase (EpiCypher) followed by addition of 2 mM CaCl_2_ and incubation for 2 hours at 4C. Stop buffer was added, and fragments were purified using the CUTANA DNA Purification Kit (Epicypher). Library preparation was done utilizing Kapa Hyperprep Kit (Roche KK8504). Libraries were sequenced on Illumina NovaSeq6000 (50 bp paired end) at Weill Cornell Medicine.

### CUT&RUN data analysis

Samples were processed using a cextflow-based pipeline available at https://github.com/michaelbale/jlabflow. Reads were trimmed using the Cutadapt wrapper trim-galore and mapped to mm10 with bowtie2 using --*very-sensitive-local* with a maximum insert size of 1000 bp. The initial bam file was then filtered for a minimum MAPQ of 30. Mitochondrial mapped reads, PCR duplicates, reads mapped within regions of a unified forbidden list from encode were removed, with additional removal of any incorrectly mated mapped reads. TMM-normalized signal tracks were visualized using deep tools bamCoverage with a calculated scaleFactor derived from the edgeR package *calcNormFactors*. For Fig. 3g and Extended Data Fig. 6j bigwigs from individual replicates were averaged together using Deeptools bigwigAverage. Differential peak analysis was carried out using DESeq2 and all peaks with an FDR ≤ .05 were called as differential.

### Stool DNA Extraction

A single fecal pellet was deposited into a Qiagen PowerBead glass 0.1 mm tube (13118-50). Using a Promega Maxwell RSC PureFood GMO and Authentication Kit (AS1600), 1 mL of CTAB buffer & 20 μL of RNAse A Solution was added to the PowerBead tube containing the sample. The sample/buffer was mixed for 10 seconds on a Vortex Genie2 and then incubated at 95°C for 5 minutes on an Eppendorf ThermoMixer F2.0, shaking at 1500 rpm. The tube was removed and clipped to a horizontal microtube attachment on a Vortex Genie2 (SI-H524) and vortexed at high-speed for 20 minutes. The sample was removed from the Vortex and centrifuged on an Eppendorf Centrifuge 5430R at 15,000 x *g* for 10 minutes. The sample was then added to a Promega MaxPrep Liquid Handler tube rack, loaded with proteinase K tubes, lysis buffer, elution buffer, 1000 µL tips, 50 µL tips, 96-sample deep-well plate, and Promega Maxwell RSC 48 plunger tips.

The Promega MaxPrep Liquid Handler instrument was programed to use 300 μL of sample and transfer all sample lysate into Promega Maxwell RSC 48 extraction cartridge for DNA extraction. Upon completion, the extraction cartridge was loaded into Promega Maxwell RSC 48 for DNA extraction & elution. DNA was eluted in 100 μL and transferred to a standard 96-well plate. DNA was quantified using Quant-iT dsDNA High Sensitivity Assay Kit using Promega GloMax plate reader on a microplate (655087).

### 16S Library Generation

Library generation follows the protocol from Earth Microbiome Project http://press.igsb.anl.gov/earthmicrobiome/protocols-and-standards/16s/.

### 16S Library Verification, Quality Check, Pooling

Amplicon libraries were washed using Beckman Coulter AMPure XP magnetic beads. Library quality & size verification was performed using PerkinElmer LabChip GXII instrument with DNA 1K Reagent Kit (CLS760673). Library concentrations were quantified using Quant-iT dsDNA High Sensitivity Assay Kit using Promega GloMax plate reader on a microplate (655087). Library molarity was calculated based on library peak size & concentration. Libraries were normalized to 2 nM using the PerkinElmer Zephyr G3 NGS Workstation (133750) and pooled together using the same volume across all normalized libraries into a 1.5 mL Eppendorf DNA tube.

### 16S Sequencing

Pooled libraries were sequenced on the Illumina MiSeq instrument at loading concentration of 7 pM with 15% PhiX, paired-end 250 using MiSeq Reagent Kit v2, 500-cycles (MS-102-2003).

### 16S Data Processing

Demultiplexed raw reads were processed using the Nextflow^31^, nf-core^32^, ampliseq pipeline^33^ version 2.5.0, with the following parameters: -profile singularity --FW_primer GTGYCAGCMGCCGCGGTAA --RV_primer CCGYCAATTYMTTTRAGTTT --dada_ref_taxonomy silva -- ignore_empty_input_files --ignore_failed_trimming --min_frequency 10 --retain_untrimmed -- trunclenf 240 --trunclenr 160. Specifically, reads were trimmed with cutadapt^34^, PhiX and quality filtering, read pair merging, and amplicon sequence variant resolution was performed with DADA2^35^. Subsequent taxonomic assignment was also performed with DADA2, using the Silva reference database^36^ version 138. Diversity analysis was performed with QIIME2^37^.

### Bulk RNA sequencing

Total RNA was isolated from 2 x 10^5^ cells untreated or butyrate-conditioned BMDMs using the NucleoSpin RNA extraction kit (Macherey-Nagel, 740902.50) according to the manufacturer’s instructions. An mRNA library was prepared by poly(A) enrichment using the Illumina TrueSeq platform. Samples were sequenced using the Illumina PE150 platform.

### Single-cell RNA sequencing and data analysis

Peritoneal lavage cells were collected as described above. Cells from different mice (two mice per condition) were stained with the following TotalSeq-A hashtag antibodies (all from BioLegend, 1 µg per 1 million cells in a final volume of 100 µl): Hashtag 1 (A0301), Hashtag 2 (A0302), Hashtag 3 (A0303), Hashtag 4 (A0304), Hashtag 5 (A0305), Hashtag 6 (A0306). Library preparation and single-cell RNA sequencing were performed by the Single Cell Research Initiative at SKI using 10X genomics Chromium Single Cell 3’ Library & Gel bead kit V3.1 according to the manufacturer’s instructions.

### Statistics and Reproducibility

Data were analyzed using GraphPad Prism v7 and v9, SPSS v.22, and R v.4.2.0. We determined significance using one of the following: unpaired two-tailed Student’s *t*-test, nonparametric Mann–Whitney U-test, one-way (Dunnett’s multiple comparisons test) or two-way ANOVA, or Fisher’s exact test. R v.4.2.0 was used for graphical and statistical analyses and the R package DESeq2 was used for differential gene expression analysis of bulk RNA-seq. Analysis of single cell RNA-seq data was performed using Python v.3.8 and R v.4.2.0. Specifically, the anaconda packages numpy, pandas, matplotlib, and scanpy as well as the R implementation of the standard Seurat v.4.0 pipeline were used in the course of data normalization, dimensionality reduction, clustering, and visualization. To facilitate these analyses, we used the web-based platform NASQAR^38^. This all-in-one platform also facilitated our ability to identify immune cell subsets by cross-referencing our data with immune cell RNA-seq performed by the Immunological Genome consortium^39^. Gene functions were determined an approach described previously^40^. In parallel, significantly differentially expressed genes were analyzed using the Molecular Signatures Database (MSigDB). Bulk RNA-seq, single cell RNA-seq, and CUT&RUN ChIP-seq data analyses and statistics are featured in Supplementary Data Tables 1-9. All biologically independent samples are included in the statistical and graphical analyses featured in the manuscript and no data was excluded. Sample sizes were not predetermined using statistical methods. Data distribution was assumed to be normal, but this was not formally tested. Statistical p values are available in Supplementary Data Table 10. Data collection and analysis were not performed blind to the conditions of the experiments. The code used in this manuscript is available upon reasonable request.

## Extended Data

## Supplementary Material

Supplementary Table 1

Supplementary Table 2

Supplementary Table 3

Supplementary Table 4

Supplementary Table 5

Supplementary Table 6

Supplementary Table 8

Supplementary Table 9

Supplementary Table 10

Table 7

## Data Availability

All data supporting the present study are available within the paper and supplementary information files. Source data are provided with this paper or can be found in GEO (GSE270512, GSE270514, and GSE270751) and SRA (PRJNA1128534).
